# Prognostic Impact of Metabolism Reprogramming Markers Acetyl-CoA Synthetase 2 Phosphorylation and Ketohexokinase-A Expression in Non-Small-Cell Lung Carcinoma

**DOI:** 10.3389/fonc.2019.01123

**Published:** 2019-11-05

**Authors:** Xueying Yang, Fei Shao, Susheng Shi, Xiaoli Feng, Wei Wang, Yalong Wang, Wei Guo, Juhong Wang, Shugeng Gao, Yibo Gao, Zhimin Lu, Jie He

**Affiliations:** ^1^Department of Thoracic Surgery, National Cancer Center/National Clinical Research Center for Cancer/Cancer Hospital, Chinese Academy of Medical Sciences and Peking Union Medical College, Beijing, China; ^2^Cancer Institute, The Affiliated Hospital of Qingdao University, Qingdao, China; ^3^Qingdao Cancer Institute, Qingdao, China; ^4^Department of Pathology, National Cancer Center/National Clinical Research Center for Cancer/Cancer Hospital, Chinese Academy of Medical Sciences and Peking Union Medical College, Beijing, China; ^5^Zhejiang Provincial Key Laboratory of Pancreatic Disease, The First Affiliated Hospital, Institute of Translational Medicine, Zhejiang University School of Medicine, Hangzhou, China

**Keywords:** metabolism reprogramming, KHK-A, ACSS2 pS659, non-small-cell lung carcinoma, prognosis, immunohistochemistry

## Abstract

**Background:** The identification of prognostic markers for non-small-cell lung carcinoma (NSCLC) is needed for clinical practice. The metabolism-reprogramming marker ketohexokinase (KHK)-A and acetyl-CoA synthetase 2 (ACSS2) phosphorylation at S659 (ACSS2 pS659) play important roles in tumorigenesis and tumor development. However, the clinical significance of KHK-A and ACSS2 pS659 in NSCLC is largely unknown.

**Methods:** The expression levels of KHK-A and ACSS2 pS659 were assessed by immunohistochemistry analyses of surgical specimens from 303 NSCLC patients. The prognostic values of KHK-A and ACSS2 pS659 were evaluated by Kaplan–Meier methods and Cox regression models.

**Results:** The expression levels of KHK-A and ACSS2 pS659 were significantly higher in NSCLC tissues than those in adjacent non-tumor tissues (*P* < 0.0001). KHK-A or ACSS2 pS659 alone and the combination of KHK-A and ACSS2 pS659 were inversely correlated with overall survival in NSCLC patients (*P* < 0.001). The multivariate analysis indicated that KHK-A or ACSS2 pS659 and KHK-A/ACSS2 pS659 were independent prognostic biomarkers for NSCLC (*P* = 0.008 for KHK-A, *P* < 0.001 for ACSS2 pS659, and *P* < 0.001 for KHK-A/ACSS2 pS659). Furthermore, the combination of KHK-A and ACSS2 pS659 can be used as a prognostic indicator for all stages of NSCLC.

**Conclusions:** KHK-A or ACSS2 pS659 alone and the combination of KHK-A and ACSS2 pS659 can be used as prognostic markers for NSCLC. Our findings highlight the important role of metabolic reprogramming in NSCLC progression.

## Introduction

Lung cancer is becoming the leading cause of cancer-related death worldwide (1). Non-small-cell lung carcinoma (NSCLC) is the most common type of lung cancer, accounting for ~85% of all cases (2). Due to the high rates of metastasis, recurrence, and drug resistance of NSCLC, its prognosis remains poor (3). There is an urgent need for novel biomarkers to identify a subset of patients with poor survival outcomes.

The reprogramming of energy metabolism is one of the hallmarks of cancer (4). During reprograming, cancer cell metabolism and other cellular activities are integrated and mutually regulated (5). Recent studies have shown that metabolic enzymes, such as ketohexokinase (KHK)-A and acetyl-CoA synthetase 2 (ACSS2), are moderated spatially and temporally in cancer cells so that these enzymes not only have changes in metabolic activities but also gain non-canonical functions (5).

KHK initiates fructose catabolism by catalyzing the transfer of a phosphate group from adenosine triphosphate (ATP) to fructose to produce AMP and fructose 1-phosphate (F1P) (6). F1P is then metabolized to dihydroxyacetone phosphate and glyceraldehyde-3-phosphate to bypass important glycolytic regulatory steps in glycolysis and enter the latter stages of glycolysis (7). KHK-A and KHK-C are splicing isoforms of KHK with one exon difference (8). KHK-C is expressed mainly in the liver, intestines, and kidney, whereas KHK-A is ubiquitously expressed at low levels (9). Although fructose can be metabolized by both KHK-C and KHK-A, the primary enzyme involved in fructose metabolism is considered to be KHK-C rather than KHK-A due to its *K*_m_ (10). We recently reported that a splicing switch from KHK-C to KHK-A occurs in hepatocellular carcinoma (HCC), leading to fructose metabolism reduction, and KHK-A phosphorylates and activates phosphoribosyl pyrophosphate synthetase 1 (PRPS1), resulting in increased *de novo* nucleic acid synthesis for HCC development (6). Under oxidative stress, KHK-A dissociates from PRPS1 and phosphorylates p62 to activate Nrf2, and activated Nrf2 induces gene expression to counteract oxidative stress and promote HCC development in mice (11). Notably, high KHK-A expression predicted a poor prognosis for HCC patients (6). Thus, KHK-A reprograms HCC cell metabolism and other cellular activities by reducing fructose metabolism and increasing *de novo* nucleic acid synthesis and the antioxidative stress response by the protein kinase activity of KHK-A. An important remaining question is whether KHK-A plays an important role in cancers other than HCC.

Histone lysine acetylation is essential for regulating chromatin architecture and promoting transcription (12). In mammalian cells, acetyl coenzyme A (acetyl-CoA) is a necessary acetyl donor for lysine acetylation and can be produced by three enzymes: ATP-citrate lyase (ACL), the pyruvate dehydrogenase complex (PDC), and acetyl-CoA synthetase (ACSS) (13–15). In nutrient-rich environments, acetyl-CoA is primarily produced by ACL (13), and growth signals promote PDC-dependent acetyl-CoA production (14). In tumors, metabolic stress frequently occurs. Our previous study revealed that AMP-activated protein kinase (AMPK) can mediate ACSS2 phosphorylation at S659 (ACSS2 pS659) to induce its nuclear translocation in a glucose-deficient environment, and the binding of ACSS2 to the promoter regions of lysosomal and autophagy genes can promote acetyl-CoA production to support histone acetylation and gene expression to promote tumor development (16). Collectively, these results suggest that ACSS2 pS659 plays an important role in tumor metabolism reprogramming through its nuclear function. However, whether ACSS2 pS659 expression is a biomarker for the clinical features and prognosis of cancer is unknown.

In this study, we examined the expression of KHK-A and ACSS2 pS659 in human NSCLC specimens and the relationship between their abundance and clinical relevance in a large cohort of surgically resected NSCLCs. We found that both KHK-A and ACSS2 pS659 are independent prognostic factors for NSCLC patients after surgery, and the combination of KHK-A and ACSS2 pS659 can be used as a prognostic indicator for all stages of NSCLC.

## Materials and Methods

### Patients and Specimens

We enrolled a total of 303 consecutive patients diagnosed with NSCLC, including 227 with lung adenocarcinoma (LUAD) and 76 with lung squamous cell carcinoma (LUSC), by pathological examination at the National Cancer Center/Cancer Hospital in Chinese Academy of Medical Sciences. Patients were diagnosed with NSCLC and were without preoperative chemotherapy, radiotherapy, and distant metastasis. All paired tumor and adjacent non-tumor tissues used in this study were collected in compliance with an informed consent policy. This study was approved by the Ethics Committee of the National Cancer Center/Cancer Hospital, Chinese Academy of Medical Sciences, and Peking Union Medical College.

We obtained clinical data by reviewing the patients' medical histories, which are summarized in Table 1. Pathological staging was assessed by the 8th edition of the American Joint Committee on Cancer/Union for International Cancer Control TNM classification system (17). We obtained completed follow-up information for all patients, and the time from the date of diagnosis to death or the last known date of follow-up was defined as overall survival (OS).

**Table 1:** Patient characteristics (*N* = 303).

**Characteristics**	**Number of patients (%)**
**Gender**	
Male	206 (68.0%)
Female	97 (32.0%)
**Age (years)**	
≤60	132 (43.6%)
>60	171 (56.4%)
**Histology**	
Adenocarcinoma	227 (74.9%)
Squamous cell carcinoma	76 (25.1%)
**T stage**	
I	63 (20.8%)
II	141 (46.5%)
III	62 (20.5%)
IV	37 (12.2%)
**Node metastasis**	
No	153 (50.5%)
**Yes**	150 (49.5%)
**TNM stage**	
I	84 (27.7%)
II	89 (29.4%)
III	122 (40.3%)
IV	8 (2.6%)

*TNM stage, tumor-node-metastasis stage*.

### Tissue Microarray and Immunohistochemistry

Prior to tissue microarray (TMA) construction, hematoxylin, and eosin (H&E)-stained slides were evaluated by two pathologists independently, and high tumor/stroma ratio areas were marked in the paraffin-embedded specimens. Two core regions (1 mm in diameter) were extracted from each marked tumor tissue and peritumoral tissue to construct the TMA slides, and consecutive sections measuring 4 μm were placed on adhesive slides (18). Immunohistochemistry (IHC) analyses of the paraffin sections were performed as previously described (19). A rabbit polyclonal antibody recognizing KHK-A was obtained from Signalway Biotechnology (Pearland, TX, Cat. #21708) and was diluted to 1:50. An anti-ACSS2 pS659 rabbit polyclonal antibody was obtained from Jiaxing Xinda Biological Technology and was diluted to 1:50. The specificities of these antibodies were previously validated (6, 16).

### Evaluation of the Immunohistochemical Findings

IHC scoring of KHK-A and ACSS2 pS659 was based on the percentage of positive cells and the staining intensity. The proportion scores were defined as follows: 0, 0% of positive cells; 1, 0–1%; 2, 2–10%; 3, 11–30%; 4, 31–70%; and 5, 71–100%. The staining intensity was rated as follows: 0, negative; 1, weak; 2, moderate; and 3, strong. A total score (range: 0, 2–8) was then obtained by combining the proportion and intensity scores, as described previously (20). IHC scoring was assessed independently by two experienced pathologists who did not know the clinical information of the patients. The cutoff values for high and low expression were based on the values of the IHC scores. In this study, the expression of KHK-A was classified as low (score ≤6) or high (score >6), and the expression of ACSS2-pS659 was classified as low (score ≤5) or high (score >5).

### Statistical Analysis

We used SPSS 21.0 (IBM, Armonk, NY) to perform statistical analyses. The association between marker expression and clinical factors was analyzed by the chi-squared (χ^2^) test. The survival analyses were performed by the Kaplan–Meier method (log-rank test). We used univariate Cox regression to calculate the risk factors for progress, and the risk factors were then included in a multivariate Cox regression model to identify the independent prognostic factors. *P* < 0.05 was considered statistically significant. All statistical tests were two-sided.

## Results

### NSCLC Specimens Have Increased KHK-A and ACSS2 pS659 Expression Levels

**Figure 1:**
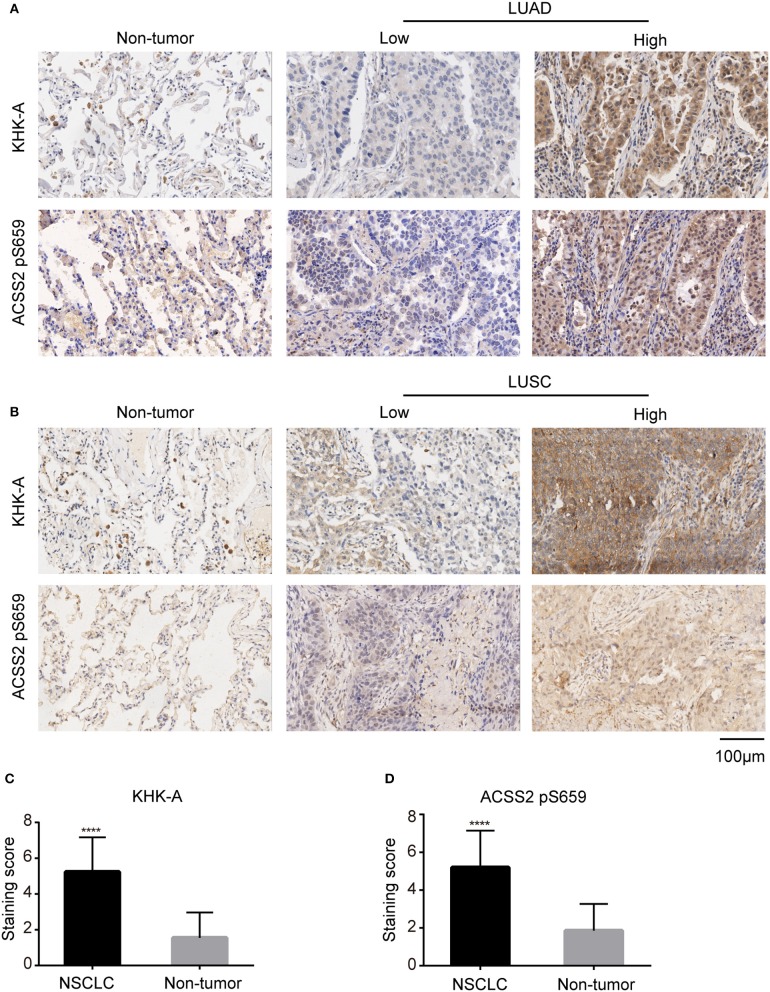
NSCLC specimens have increased KHK-A and ACSS2 pS659 expression levels. **(A,B)** Representative IHC staining of low and high expression of KHK-A and ACSS2 pS659 in NSCLC tissues and adjacent non-tumor tissues (*N* = 303). **(A)** LUAD, **(B)** LUSC. Scale bar, 200×, 100 μm. **(C,D)** The expression levels of KHK-A **(C)** and ACSS2 pS659 **(D)** in NSCLC and adjacent non-tumor tissues were compared by IHC staining. ^****^Correlation is significant at the 0.0001 level (two-tailed).

We performed immunohistochemical (IHC) staining of NSCLC specimens (*N* = 303), including LUAD (Figure 1A) and LUSC (Figure 1B) tissues. We showed that KHK-A was primary in the cytoplasm of the NSCLC cells and that ACSS2 pS659 was observed in both nucleus and cytoplasm of the NSCLC cells (Figures 1A,B). In addition, the expression levels of KHK-A and ACSS2 pS659 were significantly higher in NSCLC tissues than those in adjacent non-tumor tissues (Figures 1A–D), indicating that NSCLC specimens have increased KHK-A and ACSS2 pS659 expression levels.

### KHK-A and ACSS2 pS659 Expression Levels Are Correlated With Clinical Features in Patients With NSCLC

We examined the relationship between KHK-A and ACSS2 pS659 expression levels and clinical features in NSCLC. KHK-A expression levels were higher in LUAD than those in LUSC (*P* = 0.001). However, there were no statistical correlations between KHK-A and other clinical features, including gender, age, T classification, N classification, or TNM stage (all *P* > 0.05, Table 2). Notably, ACSS2 pS659 expression levels were higher in LUAD than those in LUSC (*P* = 0.031), and higher in female patients than in males (*P* = 0.034). In addition, high ACSS2 pS659 expression was associated with older age (*P* = 0.015) and advanced TNM stage (*P* = 0.048).

**Table 2:** Relationship of KHK-A and ACSS2 pS659 expression levels with patient characteristics.

**Characteristics**	**KHK-A expression** **level**		***P*-value**	**ACSS2 pS659 expression** **level**		***P*-value**
	**Low**	**High**		**Low**	**High**	
**Gender**						
Male	149	57	0.191	114	92	0.034
Female	63	34		41	56	
**Age (years)**						
≤60	95	37	0.504	78	54	0.015
>60	117	54		77	94	
**T stage**						
I+II	146	58	0.383	110	94	0.167
III+IV	66	33		45	54	
**Node metastasis**						
No	114	39	0.081	82	71	0.391
Yes	98	52		73	77	
**TNM stage**						
I+II	127	46	0.131	97	76	0.048
III+IV	85	45		58	72	
**Histology**						
Adenocarcinoma	147	80	0.001	108	119	0.031
Squamous cell carcinoma	65	11		47	29	

*TNM stage, tumor-node-metastasis stage*.

### KHK-A and ACSS2 pS659 Expression Levels Are Inversely Correlated With the OS of Patients With NSCLC

**Figure 2:**
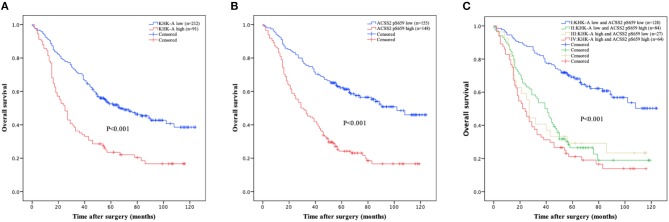
Prognostic value of KHK-A and ACSS2 pS659 expression in NSCLC. **(A,B)** The value of the IHC score was used to divide the indicated NSCLC patients into two groups with high and low levels of KHK-A **(A)** and ACSS2 pS659 **(B)** expression. Kaplan–Meier survival curves were compared using the log-rank test. All statistical tests were two-sided. Crosses represent censored data from patients who were alive at the last clinical follow-up. **(C)** IHC scores of KHK-A and ACSS2 pS659 were used to divide the NSCLC patients into four subgroups (I, KHK-A low expression, and ACSS2 pS659 low expression; II, KHK-A low expression, and ACSS2 pS659 high expression; III, KHK-A high expression, and ACSS2 pS659 low expression; IV, KHK-A high expression, and ACSS2 pS659 high expression). Kaplan–Meier survival curves were compared using the log-rank test. All statistical tests were two-sided. Crosses represent censored data from patients who were alive at the last clinical follow-up.

We next analyzed whether KHK-A and ACSS2 pS659 expression levels in NSCLC patients have any relationship with disease prognosis. As shown in Figure 2A, high KHK-A expression levels predicted a poor 5-years OS rate (high vs. low: 23.5 vs. 52.5%, *P* < 0.001). In addition, high ACSS2 pS659 expression levels were also correlated with a poor 5-years OS rate (Figure 2B; high vs. low: 24.2 vs. 62.1%, *P* < 0.001). To analyze the prognostic value of KHK-A and ACSS2 pS659 co-expression, we divided the patients into four subgroups using the IHC scores of KHK-A and ACSS2 pS659: I, KHK-A low expression and ACSS2 pS659 low expression; II, KHK-A low expression, and ACSS2 pS659 high expression; III, KHK-A high expression and ACSS2 pS659 low expression; IV, KHK-A high expression, and ACSS2 pS659 high expression. We observed that OS was significantly different among the four subgroups (Figure 2C, *P* < 0.001) and that high expression of both KHK-A and ACSS2 pS659 appeared to have the worst prognosis with the lowest survival rates (5-years OS: 21.1%). In contrast, patients with low tumor expression of both KHK-A and ACSS2 pS659 had the best prognosis with the highest OS rate (5-years OS: 69.1%).

A univariate analysis was performed to investigate the risk factors for OS. As shown in Table 3, T stage, node metastasis, histology, KHK-A, and ACSS2 pS659 were significantly associated with OS (*P* < 0.05). Statistically significant variables were added to multivariate survival analyses. In the Cox regression model, node metastasis, histology, KHK-A expression, and ACSS2 pS659 levels were independent prognostic factors of OS [hazard ratio (HR) = 2.003, 95% confidence interval (95% CI) = 1.486–2.700 for node metastasis; HR = 0.65, 95% CI = 0.434–0.795 for histology; HR = 1.533, 95% CI = 1.120–2.099 for KHK-A expression; and HR = 2.313, 95% CI = 1.687–3.172 for ACSS2 pS659]. To evaluate the prognostic value of combined KHK-A and ACSS2 pS659 expression in NSCLC, we considered the expression of KHK-A and ACSS2 pS659 as a single factor for a separate multivariate analysis. Table 4 shows that the combination of KHK-A and ACSS2 pS659 was an independent predictor of OS (HR = 2.803, 95% CI = 1.920–4.094 for II vs. I; HR = 2.319, 95% CI = 1.366–3.936 for III vs. I; and HR = 3.587, 95% CI = 2.413–5.331 for IV vs. I).

**Table 3:** Univariate and multivariate analyses of overall survival for 303 NSCLC.

**Factor**	**Univariate** ***P*-value**	**Multivariate**		
		**Hazard ratio**	**95% CI**	***P*-value**
Gender (female vs. male)	0.930			NA
Age (>60 vs. ≤60)	0.065			NA
T stage (I vs. II vs. III vs. IV)	0.017			0.811
Node metastasis (yes vs. no)	<0.001	2.003	1.486–2.700	<0.001
Histology (squamous vs. adenocarcinoma)	0.001	0.65	0.434–0.975	0.037
KHK-A (high vs. low)	<0.001	1.533	1.120–2.099	0.008
ACSS2 pS659 (high vs. low)	<0.001	2.313	1.687–3.172	<0.001

*CI, confidence interval; NA, not adopted*.

**Table 4:** Univariate and multivariate analyses of overall survival for 303 NSCLC.

**Factor**	**Univariate** ***P-*value**	**Multivariate**		
		**Hazard ratio**	**95% CI**	***P*-value**
Gender (female vs. male)	0.930			NA
Age (>60 vs. ≤60)	0.065			NA
T stage (I vs. II vs. III vs. IV)	0.017			0.823
Node metastasis (yes vs. no)	<0.001	1.953	1.446–2.637	<0.001
Histology (squamous vs. adenocarcinoma)	0.001	0.661	0.441–0.992	0.046
Combination of KHK-A and ACSS2 pS659	<0.001			<0.001
II vs. I	<0.001	2.803	1.920–4.094	<0.001
III vs. I	<0.001	2.319	1.366–3.936	0.002
IV vs. I	<0.001	3.587	2.413–5.331	<0.001

*CI, confidence interval; NA, not adopted; I, KHK-A^Low^/ACSS2 pS659^Low^; II, KHK-A^Low^/ACSS2 pS659^High^; III, KHK-A^High^/ACSS2 pS659^Low^; IV, KHK-A^High^/ACSS2 pS659^High^*.

**Figure 3:**
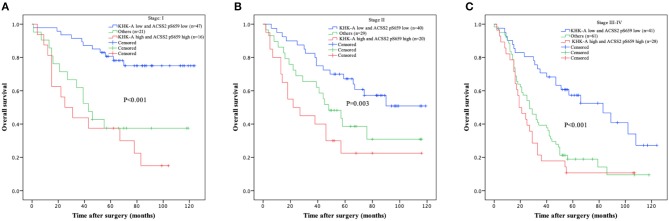
Prognostic value of combined KHK-A and ACSS2 pS659 expression in NSCLC based on TNM stage. **(A–C)** IHC scores of KHK-A and ACSS2 pS659 were used to divide the NSCLC patients into three subgroups (KHK-A low expression and ACSS2 pS659 low expression, KHK-A high expression and ACSS2 pS659 high expression, and others: KHK-A low expression and ACSS2 pS659 high expression or KHK-A high expression and ACSS2 pS659 low expression) in stage I **(A)**, stage II **(B)**, and stage III–IV **(C)**. Kaplan–Meier survival curves were compared using the log-rank test. All statistical tests were two-sided. Crosses represent censored data from patients who were alive at the last clinical follow-up.

Furthermore, we analyzed the relationship between combination of KHK-A and ACSS2 pS659 and prognosis from TNM stage I to IV. Low combined values of KHK-A and ACSS2 pS659 expression were associated with improved OS in stages I (Figure 3A, *P* < 0.001), II (Figure 3B, *P* = 0.003), and III–IV (Figure 3C, *P* < 0.001). These results indicate that the combined expression of KHK-A and ACSS2 pS659 is inversely correlated with OS in all stages of NSCLC.

## Discussion

Although tremendous clinical success in the treatment of NSCLC during the past few decades has been achieved, the prognosis of patients with NSCLC remains unsatisfactory. We still need to explore biological characteristics that can reflect NSCLC behavior. In this study, we showed that the expression levels of metabolism reprogramming marker KHK-A and ACSS2 pS659 were significantly higher in NSCLC tissues than those in adjacent non-tumor tissues, and KHK-A expression levels were higher in LUAD than those in LUSC. In addition, increased ACSS2 pS659 expression was correlated with older age, advanced TNM stage, LUAD, and female patients. Of note, we showed that high expression levels of KHK-A and ACSS2 pS659 were correlated with reduced OS in NSCLC. In the multivariate analysis, both KHK-A and ACSS2 pS659 expression levels were identified as independent prognostic biomarkers for NSCLC. These findings suggest that KHK-A and ACSS2 pS659 are potential prognostic indicators for NSCLC.

Our previous work demonstrated that KHK-A and ACSS2 are two key metabolic enzymes that have important roles in tumor development. KHK-A can act as a protein kinase to directly phosphorylate PRPS1 to increase *de novo* nucleic acid synthesis for hepatocellular tumorigenesis (6). In addition to its role in nucleic acid synthesis, KHK-A phosphorylates p62, leading to the activation of Nrf2 and the expression of its downstream genes to counteract oxidative stress and support HCC development (11). Reports on the role of ACSS2 expression in cancer have been controversial. High ACSS2 expression predicted a poor prognosis in patients with renal cell carcinoma and bladder cancer (21, 22), whereas low ACSS2 expression predicted a poor prognosis in gastric cancer and hepatocellular carcinoma (23, 24). Our previous study showed that the phosphorylation of ACSS2 at S659 rather than its expression level is critical for GBM cells to counteract energy stress. S659-phosphorylated ACSS2 translocates to the nucleus, leading to the binding to downstream genes and acetyl-CoA production, which induces histone acetylation and gene expression for glioma development (16, 25). Although KHK-A and ACSS2 pS659 have been studied in HCC and GBM, respectively, their roles in NSCLC had not yet been explored. We showed here that the combination of KHK-A and ACSS2 pS659 was an independent prognostic biomarker for NSCLC and was inversely related with OS in all stages of NSCLC. Given that multiple randomized clinical trials have suggested that postoperative NSCLC patients with a poor prognosis are more likely to benefit from adjuvant therapy (26), the combination of KHK-A and ACSS2 pS659 expression levels can be taken into consideration for decisions regarding the use of adjuvant therapy for NSCLC, especially in the early stage.

## Conclusions

In summary, KHK-A or ACSS2 pS659 alone and the combination of KHK-A and ACSS2 pS659 can be used as prognostic markers for NSCLC. Our findings highlight the importance of metabolic reprogramming in the clinical behavior of NSCLC and reveal that KHK-A and ACSS2 pS659 can be targeted for NSCLC treatment.

## Data Availability Statement

All datasets generated for this study are included in the article/supplementary material.

## Ethics Statement

All samples used in this study were collected with signed informed consent, and this study was approved by the Ethics Committee of the National Cancer Center/Cancer Hospital, Chinese Academy of Medical Sciences, and Peking Union Medical College.

## Author Contributions

XY, YG, ZL, and JH conceived and designed the study and carried out data interpretation. XY wrote the manuscript with help from YG, ZL, and JH. SG, WG, and JW collected tissue samples. XY, WW, and JW performed the immunohistochemical assays. SS and XF evaluated and scored the staining. XY, FS, and YW acquired data and performed the statistical analysis. All authors read and approved the final manuscript.

### Conflict of Interest

ZL owns shares in Signalway Biotechnology (Pearland, TX), which supplied the rabbit antibody that recognizes KHK-A. ZL's interest in this company had no bearing on its being chosen to supply this reagent. The remaining authors declare that the research was conducted in the absence of any commercial or financial relationships that could be construed as a potential conflict of interest.
